# Intolerance to Multiple P2Y12 Inhibitors Following Percutaneous Coronary Intervention

**DOI:** 10.7759/cureus.13574

**Published:** 2021-02-26

**Authors:** Mina Shnoda, Kinan Kassar, Deanna L Huffman, Vijaya Sanikommu, Indu Poornima

**Affiliations:** 1 Internal Medicine, Allegheny Health Network, Pittsburgh, USA; 2 Cardiovascular Disease, Allegheny Health Network, Pittsburgh, USA; 3 Cardiology, Allegheny Health Network, Pittsburgh, USA

**Keywords:** p2y12 inhibitors, clopidogrel hypersensitivity, multiple drug intolerance, percutaneous coronary intervention

## Abstract

Dual antiplatelet therapy (DAPT), defined as administration of a P2Y12 receptor inhibitor (clopidogrel, prasugrel, or ticagrelor) and aspirin, is recommended after percutaneous coronary intervention. We describe a case of a 50-year-old gentleman with intolerance to the three previously mentioned P2Y12 inhibitors following the placement of a drug-eluting stent to the left anterior descending artery. To our knowledge, based on a thorough review of the literature, this is the second case reporting a similar medical dilemma. We have discussed the multidisciplinary approach implemented to overcome this clinical challenge, which involved the use of clopidogrel with simultaneous administration of a six-day course of oral steroids.

## Introduction

Patients with hypersensitivity to any single P2Y12 inhibitor following percutaneous coronary intervention (PCI) are commonly managed by shifting to a different P2Y12 inhibitor [1,2]. We present a case of a post-PCI patient with intolerance to multiple P2Y12 inhibitors and the multi-disciplinary approach used to overcome such a clinical challenge. 

## Case presentation

**Figure 1:**
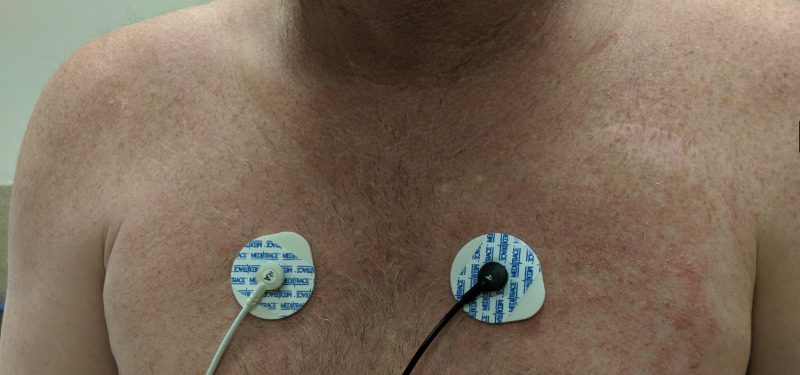
Rash predominantly affecting patient’s chest

A 50-year-old male with past medical history of ischemic heart disease and prasugrel hypersensitivity in the form of generalized rash underwent elective PCI with one drug-eluting stent (DES) to the left anterior descending artery (LAD). He was subsequently started on dual antiplatelet therapy with aspirin 81 mg daily and ticagrelor 90 mg twice a day. Two days following discharge, the patient was complaining of severe shortness of breath with minimal exertion, and occasionally at rest. He denied any fevers, chills, productive cough, orthopnea, paroxysmal nocturnal dyspnea or lower limb edema. The patient’s dyspnea was heavily investigated with basic labs, cardiac biomarkers, imaging of the chest, and repeat transthoracic echocardiogram. All results came back within normal limits. The patient’s dyspnea was deemed secondary to ticagrelor use and the drug was discontinued, with subsequent improvement of his symptoms. Given his history of prasugrel hypersensitivity and current intolerance to ticagrelor, the patient was started on clopidogrel. Three days later, he presented to the emergency department (ED) with a rash that started on his chest and spread to his arms and face (Figure 1).

The patient was diagnosed with cutaneous hypersensitivity to clopidogrel. Given the patient’s need for dual antiplatelet therapy and previously documented prasugrel hypersensitivity as well as intolerance to ticagrelor, shifting to any of them was not an option. 

Reviewing the literature, three management strategies have been described for clopidogrel hypersensitivity, including switching to another agent, implementing a desensitization protocol, and a six-day course of steroids without clopidogrel discontinuation. A shared decision was made to implement the latter approach however with a slow taper over a three-week period. The patient was started on oral prednisone 30 mg twice a day for five days, followed by a decrease of 5 mg/day every three days for 15 days. On his one-week follow-up, the patient was well-tolerating clopidogrel with complete resolution of his symptoms and no significant side effects from steroids. The patient showed no side effects related to steroids on his one-month follow-up. 

## Discussion

The current American College of Cardiology Foundation/American Heart Association Task Force on Practice Guidelines/Society for Cardiovascular Angiography and Interventions (ACCF/AHA/SCAI) guidelines recommend at least 12 months of dual antiplatelet therapy after DES implantation. Clopidogrel is widely used after DES placement to protect against stent thrombosis.

Clopidogrel hypersensitivity incidence is estimated at 6% and results in premature drug discontinuation in 1.5% of patients [3]. It is believed to be secondary to lymphocyte-mediated delayed hypersensitivity and presents clinically in one of three forms. The first and most common presentation is in the form of a generalized, pruritic rash affecting the trunk with or without the involvement of upper or lower extremities; second, a rash limited to the localized area involving the neck, face, back, axilla, palms, or soles. The third and least common is in the form of angioedema [1]. A skin biopsy was not obtained in our case study as the patient opted against any invasive investigation. 

Several management strategies for clopidogrel hypersensitivity have been described in the medical literature, including shifting to another P2Y12 inhibitor agent or clopidogrel desensitization [1,2]. However, there is an increased likelihood of cross-reactivity between clopidogrel, prasugrel, and ticlopidine attributed to their similar thienopyridine structure [4,5], whereas ticagrelor is a nonthienopyridine P2Y12 inhibitor and preferred over other agents for the theoretical less risk for cross-reactivity with clopidogrel [6]. Ticagrelor use is associated with mild-moderate dyspnea in approximately 14% of patients, with 0.9% of those requiring discontinuation of the drug [7].

The current case report describes a patient with a recently placed DES presenting with hypersensitivity reaction to clopidogrel. His past medical history was further complicated by documented hypersensitivity to prasugrel and severe dyspnea associated with the use of ticagrelor. Intolerance to the three P2Y12 inhibitors has seldom been reported in the literature [8] and represents a serious management dilemma in post-PCI patients.

## Conclusions

In our case study, clopidogrel hypersensitivity was successfully managed without discontinuation of clopidogrel. The administration of a six-day course of oral steroids resulted in complete resolution of symptoms. The successful management supports the conclusion previously drawn by the study done by Cheema and his colleagues, demonstrating that treatment with a short course of oral steroids can be safe and highly effective in the management of cutaneous clopidogrel hypersensitivity, especially in the absence of alternative solutions.
